# Management considerations for establishing a coastal acidification monitoring system from U.S. Coastal Acidification Networks

**DOI:** 10.1007/s10661-025-14434-3

**Published:** 2025-08-07

**Authors:** Elizabeth K. Wright-Fairbanks, Natalie Lord, Darcy Dugan, Kaitlin Goldsmith, Emily R. Hall, Alex Harper, Janet J. Reimer, Samantha Siedlecki, Elizabeth J. Turner, Jennifer Vreeland-Dawson, Kirstin Wakefield, Kimberly K. Yates

**Affiliations:** 1 University Corporation for Atmospheric Research, Boulder, CO USA; 2 National Oceanic and Atmospheric Administration, Silver Spring, MD 20910 USA; 3 University of New Hampshire, Durham, NH USA; 4 Alaska Ocean Observing System, Anchorage, AK USA; 5 Alaska Ocean Acidification Network, Anchorage, AK USA; 6 Mote Marine Laboratory, Sarasota, FL USA; 7 Southeast Ocean and Coastal Acidification Network, Sarasota, FL USA; 8 California State Polytechnic University, Humboldt, CA USA; 9 California Current Acidification Network, San Diego, CA USA; 10 California Sea Grant, San Diego, CA USA; 11 Mid-Atlantic Regional Council On the Ocean, Washington, DC USA; 12 Mid-Atlantic Coastal Acidification Network, Washington, DC USA; 13 University of Connecticut, Groton, CT USA; 14 Northeast Coastal Acidification Network, Portland, ME USA; 15 Gulf of America Coastal Acidification Network, College Station, TX USA; 16 Mid-Atlantic Regional Coastal Ocean Observing System, Washington, DC USA; 17 Gulf of America Coastal Ocean Observing System, College Station, TX USA

**Keywords:** Ocean acidification, Monitoring network, Ocean observing, Coastal acidification

## Abstract

Ocean acidification (OA), caused by the uptake of anthropogenic carbon dioxide, is a concern for ocean resource users in coastal regions where the phenomenon is compounded by variable processes. Sustained OA monitoring systems are critical for characterization of baseline ocean conditions and identification of changes and impacts to coastal ecosystems and communities. Establishing an OA monitoring network is best accomplished through iterative planning, sustained funding, and comprehensive understanding of the coastal system. This paper offers decision-making considerations for entities interested in establishing a local to regional scale OA observing system. Such considerations include which carbon system parameters can be measured in each system, which sensors and platforms will provide applicable information for interested partners, and best practices for observing data management. Because every region faces unique circumstances, we present context-specific examples of effective decision-making processes from established U.S. OA observing networks. These regional case studies offer information on specific scientific questions, observing techniques, and methodology employed to establish and manage OA observations in the coastal zone.

**Supplementary Information:**

The online version contains supplementary material available at 10.1007/s10661-025-14434-3.

## Introduction

Ocean acidification (OA), caused by uptake of anthropogenic carbon dioxide from the atmosphere and compounded by complex and variable coastal processes, is an established concern for coastal managers, stakeholders, and decision makers nationwide. Ocean acidification has caused declines in global ocean pH and carbonate saturation states (Ω), with lasting implications for socioeconomically vital fisheries and coastal ecosystems (Doney et al., 2020; Subcommittee on Ocean Science and Technology Committee on Environ., 2023). The impacts of acidification are expected to continue into the future, with global surface ocean pH declines of 0.29 (constituting a 100% increase in acidification) projected by the end of the twenty-first century (Intergovernmental Panel on Climate Change [IPCC], 2019). While OA is a global phenomenon, conditions vary over geographies and the severity and rates of change are impacted by an array of local drivers. Local variability necessitates high resolution, geographically focused carbonate system monitoring in the open ocean, coastal zone, and nearshore.

The 2023 federal Interagency Working Group on Ocean Acidification (IWG-OA) vulnerability assessment identified key gaps in understanding of local conditions and processes, including the need for sustained monitoring systems targeting regional interests (Subcommittee on Ocean Science and Technology, 2023). Identified monitoring needs included (1) prioritizing measurements at scales beneficial to coastal decision makers, (2) conducting subsurface monitoring in fishery zones, (3) establishing estuarine monitoring systems, and (4) strengthening partnerships with community-based water quality monitoring programs to enhance local capabilities. The IWG-OA vulnerability assessment also highlighted the critical role of Coastal Acidification Networks (CANs) in providing regionally-specific information on OA vulnerability to a national audience. The CANs engage ocean users interested in addressing ocean and coastal acidification, and play a critical role in supporting regional monitoring, community engagement, state action planning, and the identification of mitigation and adaptation strategies. They are uniquely situated to provide expertise on strategies to establish and improve coastal acidification monitoring networks in the face of continued ocean change.

Approaches for OA observing include deploying sensors to measure pH, total alkalinity (TA), the partial pressure of carbon dioxide (*p*CO_2_), or dissolved inorganic carbon (DIC) along with temperature (T), salinity (S), dissolved oxygen (DO), and nutrient levels on a variety of platforms. Observing platforms range from in situ moorings to ship-based and uncrewed systems, as well as community sampling operations and hyper-local monitoring from docks and piers. Each approach has its own benefits and challenges with respect to installation, maintenance, cost and sensor precision.

Keeping in mind coastal managers, researchers, community members, and industry groups interested in OA monitoring, we provide examples from around the U.S. of OA observing systems designed for specific science questions. Some examples will highlight successful partnerships between tribal and U.S. federal entities that have supported OA monitoring in the recent past. Tribes have generations of experience observing ocean change in regions including Alaska and the Pacific Northwest, and play a unique and important role in the observing system (Wyllie de Echeverria & Thornton, 2019). Monitoring needs vary across geographies. Here, we provide general information on instrumentation and data archival alongside specific case studies showcasing how systems are set up in various regions and ecosystems. The information presented here is meant to aid coastal managers in OA observing decision-making processes.

## Methods

Coastal Acidification Networks, sponsored by NOAA’s Ocean Acidification Program (NOAA OAP), are led by regional experts in coastal acidification observing and science. Beginning in 2022, CAN leaders came together to review the state of understanding on needs for establishing ocean acidification monitoring networks, with a specific focus on coastal and estuarine monitoring conducted by coastal resource managers. Bringing together a review of the available literature on OA observing sensors, platforms, and data repositories with their own expertise in OA monitoring, the CAN leaders designed a “how-to” guide for establishing an OA monitoring network in the coastal zone. Highlighting the application of this guide, CAN leaders compiled case studies on effective OA monitoring networks from their regions. These case studies provide information on specific scientific questions, observing techniques, and methodology employed to establish and manage OA observing systems and address real-world ocean observing problems.

## Results

**Fig. 1:**
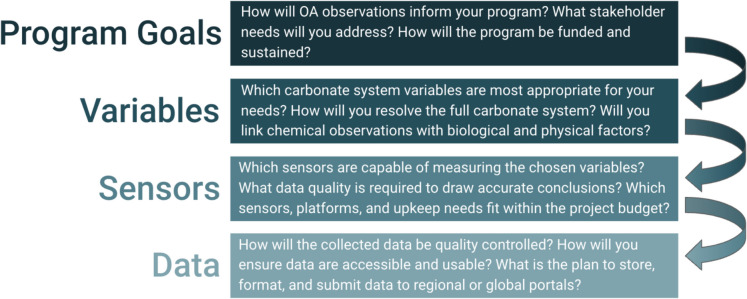
Flow chart showing suggested considerations for establishing a regional or local acidification monitoring network

Coastal managers interested in establishing an OA observing network may consider multiple variables to create a thoughtful, impactful, and scientifically sound system for their regional data users (Fig. 1). The first step in establishing an OA observing network is to identify specific goals for the program and the intended impact of any actions on regional populations. Next, specific variables and sensors can be assessed for their applicability to the program goals. Lastly, creating an informed data management plan ahead of deployment will ensure the network is impactful to coastal communities by providing findable, accessible, interoperable, and reusable (FAIR; Wilkinson et al., 2016) data and information to support resource management decisions.

### Program goals

Coastal managers can work with interested parties in the region to understand community needs, goals, and questions that will be addressed by the collected observations with focus on a specific end goal, whether that goal is information delivery, product development, or informing regional policy. The observing network can best meet the program’s goals by addressing the needs of regional communities and answering specific scientific questions that will provide actionable insights to the region. Additionally, it is imperative to have a plan in place for the funding and sustenance of the network. Many goals can only be achieved through long-term, continuous observing efforts.

### Variables and empirical relationships

There are several important considerations in determining the best measurements for an OA monitoring system. These considerations include, but are not limited to, assessing which variables and sampling frequencies are most appropriate to measure for program-specific needs, how the full carbonate system will be resolved, whether chemical observations need to be linked with biological or physical factors, and how site-specific characteristics will impact network establishment (Dickson et al., 2007; Pimenta & Grear, 2018; Riebesell et al., 2011). Considerations for a preliminary assessment of each sampling site include investigating the tidal and salinity ranges; diurnal fluctuations; biological processes, including the consumption and respiration of carbon and oxygen; and the presence of multiple stressors such as nutrient runoff, low oxygen conditions, and upwelling.

The four main carbonate chemistry variables that are measured to assess acidification are TA, DIC, *p*CO_2_, and pH (total and National Bureau of Standards [NBS] scales). These four parameters are chemically linked. To establish a rigorous program to determine the state of acidification and drivers of its variability, at least two of the four variables must be measured simultaneously along with T and S (Dickson et al., 2007). Often, DO and dissolved nutrient samples are collected alongside OA measurements through water quality programs in inter-coastal and near-shore waters (e.g., National Science Foundation Long Term Ecological Research (LTER), National Oceanic and Atmospheric Administration National Estuarine Research Reserves (NERRS), or Environmental Protection Agency National Estuary Programs (NEPs)). Considerations for variable selection include the required accuracy and precision of measurements, access to historical observing data in the region, team member expertise, fieldwork duration, access to hazardous chemical disposal services, and cost.

#### ***pH (pH***_***T***_***/pH***_***NBS***_***)***

Seawater pH is most commonly measured using the pH_T_ (total hydrogen scale; H^+^ concentrations) or pH_NBS_ (National Bureau of Standards scale; H^+^ activity) scale. These two scales can be measured directly (spectrophotometrically for pH_T_ and potentiometrically for pH_NBS_) or calculated using at least two of the other carbonate chemistry parameters. NBS pH measurements are collected as part of water quality monitoring programs throughout many NERRs, NEPs, and state and local programs. In general, the chosen pH instrument should have high enough precision to accurately measure daily variability in the system.

#### Partial pressure of carbon dioxide

The partial pressure of CO_2_ (*p*CO_2_) can be measured directly through the use of underway systems or moored systems (e.g., DeGrandpre et al., 1995; Pierrot et al., 2009; Tabacco et al., 1999; Takeshita et al., 2018). These systems are typically autonomous or semi-autonomous; however, they require data processing to account for temperature fluctuation, pressure changes, and accumulation of organic matter (Intergovernmental Oceanographic Commission, 2004). Furthermore, the equipment necessary to conduct continuous *p*CO_2_ monitoring can be expensive, and a professional trained in quantitative data analysis is required for data processing.

#### Total alkalinity and dissolved inorganic carbon

Total alkalinity (TA) is generally considered to be the primary base property for buffering seawater against acidification (Middelburg et al., 2020). Gran titration methods are the best practice for measuring TA in oceanic and coastal waters (Gran, 1952). In many traditional and wide-ranging water quality monitoring programs, TA is analyzed using standard methods and is reported as milligram per liter CaCO_3_ (Lipps et al., 2023). Specialized seawater methods (Dickson et al., 2007) provide higher precision and accuracy and are typically reported as micromole per kilogram.

The dissolved inorganic carbon (DIC) pool is made up of carbonic acid (H_2_CO_3_), carbonate (CO_3_^2−^), carbon dioxide (CO_2_), and bicarbonate (HCO^3−^). Due to Henry’s Law impacting the amount of CO_2_ dissolved in seawater, DIC is greatly influenced by temperature. DIC is commonly quantified via a non-dispersive infrared detector.

To avoid interference from suspended particulate matter, whether there are carbonate minerals, organic particles, and/or biota present in coastal waters, it may be necessary to filter or treat water samples before TA and DIC analysis. Commonly, both TA and DIC samples are treated with saturated mercuric II chloride solution to remove biotic influence. Often a barrier to collecting DIC and TA samples, mercuric II chloride requires hazardous waste disposal permits and training. However, if samples are filtered and stored in a cool laboratory environment or transported in coolers with ice packs, they are stable up to one week (Moore et al., 2024). The ability to store and transport discrete samples without the use of mercuric II chloride opens additional sampling opportunities, including citizen science initiatives (Gassett et al., 2021) and field validation programs.

#### CO2SYS

Any two of the four measured carbonate system parameters, in addition to pressure, temperature, and salinity, can be used to estimate the remaining carbonate system parameters including calcite and aragonite saturation states (Ω). Therefore, the impact of ocean acidification monitoring systems is strengthened when carbonate system measurements are coupled with temperature and salinity measurements. These variables are used as inputs in the CO2SYS software program to resolve the full carbonate system. CO2SYS is open source and available in Excel, MATLAB, Python, and R (Gattuso et al., 2023; Humphreys et al., 2022; Sharp et al., 2020). Numerous studies have highlighted the utility of CO2SYS and have used CO2SYS as a quality assurance tool when multiple monitoring or sampling methods are used, ensuring that all methods accurately produce the same results (Lueker et al., 2000; Millero, 2010; Patsavas et al., 2015; Reimer et al., 2017b).

#### Empirical relationships to estimate OA

Algorithms and empirical models developed globally and regionally can estimate OA conditions from observational data (e.g., T, S, DO, pH, and *p*CO_2_; Juranek et al., 2009; Alin et al., 2012; Williams et al., 2016; McGarry et al. 2021; Carter et al., 2021). These basic hydrographic measurements can be more accessible to coastal managers, making algorithms key to begin observing OA variability. In addition, they can be used to extend existing historical time series into undersampled carbon variables, underscoring the utility of existing hydrographic information to support regional OA observing efforts. Local relationships are necessary as algorithms often differ between locations (McGarry et al. 2021).

#### *OA-relevant biological monitoring *in situ

Research on the effects of OA on biological processes and ecosystems has increased over the last decade and has focused mainly on calcifying species. The results of this work are an important aspect of coastal and marine models examining the impacts of acidification (Doo et al., 2020; Garrard et al., 2013). Due to the cost and complexity of observing these processes in the field, many of these biological studies have occurred in a laboratory setting. However, co-locating biological and chemical observations in situ is critical in understanding impacts of OA on our coastal ecosystems and an avenue for continued research.

There are achievable biological observations that can be evaluated to observe impacts of OA. Chlorophyll *a*, organic nutrients, and dissolved oxygen are examples of biologically-mediated parameters that are often included in observing systems to indicate changes in the biological system that can be compared to OA conditions to derive patterns of chemical and biological relationships. Planktonic species composition is also linked to ecosystem health and therefore is critical in evaluating ecosystem-wide responses to OA. Observations of OA indicator species can be used to identify when and where coastal ecosystems may be under environmental stress.

### Sensors

Each carbonate system or OA variable can be measured by a variety of sensors and platforms that can be tailored to a program’s needs and abilities. Choosing a carbonate system measurement technique depends on the resolution needed to accurately and precisely quantify changes relevant to answering program-specific management questions. It is important to balance data quality with project budgets for sensor purchasing, platform costs, and system maintenance. Detailed best practice guidance for measurement of carbonate system variables is available from Dickson et al. (2007). Links to standard operating procedures (SOPs) and other methods are provided in Table S-1. Best practice guidance for designing OA experiments can be found in Riebesell et al. (2011).

Changes in carbonate system variables caused by uptake of excess CO_2_ from the atmosphere to seawater occur relatively slowly, over decades; thus, detection of small changes in the carbonate system (pH, *p*CO_2_, TA, and DIC) over time periods shorter than decades requires highly accurate and precise analytical techniques that provide “climate-quality” measurements. Climate-quality measurements must be able to estimate a change in the carbonate ion concentration with a relative standard uncertainty of 1% (Newton et al., 2015). This corresponds to an uncertainty of 0.003 in pH; of 2 μmol kg^–1^ in total alkalinity and total dissolved inorganic carbon; and a 0.5% in the partial pressure of carbon dioxide. These measurements are achievable by few high-accuracy instruments in the laboratory setting (Newton et al., 2015).

Variation of the near-coastal carbonate system due to processes like river runoff, upwelling, and eutrophication typically occurs on shorter timescales (hours to years) and with higher magnitude relative to changes in the open ocean. Therefore, detection of these changes can typically be quantified using analytical techniques with higher uncertainty than is needed for open ocean measurements. These measurements with higher uncertainty are considered “weather-quality,” are generally achievable by most sensors and laboratories (Newton et al., 2015), and are therefore more likely to be accessible to coastal managers designing OA monitoring systems. Weather-quality measurements must be able to measure carbonate ion concentration with a relative uncertainty of 10% (Newton et al., 2015). This corresponds to an uncertainty of 0.02 in pH, 10 μmol kg^–1^ in TA and DIC, and 2.5% in *p*CO_2_.

Questions related to the occurrence, scale, and trends of OA, and its drivers, processes, and ecosystem impacts, can typically be answered through a combination of time series measurements. High temporal resolution time series measurements, with frequencies ranging from minutes to hours, can be achieved using data-logging sensors and real-time (telemetered) sensors on fixed platforms. High spatial resolution monitoring can be achieved through field campaigns and cruises designed to collect discrete water samples across geographic areas of interest, or through deployment of underway sensors on ships or autonomous vessels. Sensors for measurement of pH, *p*CO_2_, and related variables are commercially available and examples are listed in Table S-2. Examples of monitoring program applications of sensor and discrete sampling techniques are listed in Table S-3. Best practice guidance for assessing trends of ocean acidification time series is available in Sutton et al. (2022).

Funding for chemistry monitoring efforts is often leveraged and site-specific opportunities can be considered. For example, if there is existing infrastructure for moored autonomous sensors, adding one of the aforementioned sensors for pH and/or *p*CO_2_ could be beneficial. Sensor selection will depend on calibration needs, data storage capacity, and biofouling modifications. If there is an established water quality sampling program, DIC/TA sampling could simply be added to routine sample collection. Laboratory methods for assessing DIC and TA are well established (Dickson et al., 2007) and some laboratories offer pay-per-sample services that lower the barrier to entry.

### Data

Data Quality Assurance/Quality Control (QA/QC) and archival are critical steps in establishing an effective OA observing network. Public access to QA/QCed observational data enhances the OA community’s opportunities to contribute to data synthesis efforts and data-driven decision-making. Many coastal water quality monitoring programs are required to submit their data to specific repositories based on funding agency, umbrella network requirements, or agency best practices. Therefore, we do not recommend any one repository, but suggest that managers consider the interoperability and accessibility of different systems available to them. Submission to publicly accessible, visible, and trusted data portals is an important first step in data management. Table 1 outlines examples of OA data repositories at the international, U.S. national, regional, state, and local levels.

**Table 1:** Example data repositories and their relevant types of data and audience/scale

Data repository audience/scale	Example repositories	Links	Data ingested
International	SOCAT	https://www.socat.info/	Climate quality
International	GOA-ON	https://portal.goa-on.org/	Weather and climate quality
National	NCEI OCADS	https://www.ncei.noaa.gov/products/ocean-carbon-acidification-data-system	Climate quality
Regional	IOOS Data portals	https://ioos.noaa.gov/data/access-ioos-data/	Weather and climate quality
State	California OAH Portal	https://oah.caioos.org	Weather and climate quality
Local	Eyes on the Bay	https://eyesonthebay.dnr.maryland.gov/	Weather and climate quality

The best data archival system for any particular observational data will depend on the type of data collected and the primary audience for the data. An initial step in identifying the appropriate data repository is ensuring it is capable of ingesting the data being collected, whether it is discrete carbonate chemistry data, continuous carbon data at a fixed point over time, spatial cruise data, or biological data. All data should be accompanied by clear and concise metadata that includes any corrections made during QA/QC processes along with information about when, where, and how the data were collected. Some repositories will require certifications for data to be accepted, such as IOOS data portals which require ISO Certification.

In addition to ensuring public access to QA/QCed observational data and selecting the appropriate data repository based on the type of data and audience, there are several other important considerations to enhance data synthesis and facilitate decision-making in the field of ocean acidification.

1. Data Interoperability: To promote data synthesis and integration across different datasets and platforms, it is crucial to establish common data standards and formats. Implementing standardized data formats, such as those provided by the Climate and Forecast conventions, can facilitate data interoperability and enhance the ability to combine and analyze diverse datasets.
2. Data Sharing Policies: Clear and transparent data sharing policies can encourage researchers, institutions, and organizations to contribute their observational data to the public domain. Open data policies, where researchers are encouraged or required to share their data openly, can foster collaboration, accelerate scientific progress, and enable data-driven decision-making.
3. Data Integration and Model-Data Fusion: When combined, observational data and numerical models can enhance our understanding of OA processes and improve predictive capabilities. Integration methods, such as data assimilation techniques, can be used to merge observational data with model simulations, reducing uncertainties and improving the accuracy of model predictions.
4. Data Visualization and User-Friendly Interfaces: Providing user-friendly data visualization tools and interfaces can make complex data more accessible to a wider range of stakeholders, including policymakers, resource managers, and the general public. Interactive visualizations, maps, and dashboards can facilitate data exploration, interpretation, and communication of OA-related information. Several of the data portals listed above provide data visualization already.
5. Collaborative Networks and Data Harmonization: Collaboration among researchers, institutions, and agencies at regional, national, and international levels is essential to foster data sharing, harmonization, and standardization efforts. Collaborative networks, such as the regional CANs, can support the development of best practices, data quality control protocols, and data synthesis initiatives.
6. Long-term Data Archiving and Preservation: Long-term archival and preservation of OA observational data is crucial for its continued availability and reusability. Data repositories and archival systems that implement robust data management practices, including regular backups, metadata preservation, and data versioning safeguard the integrity and accessibility of the data over time.

By considering these additional factors, OA observing system managers can contribute to data synthesis efforts and support informed decision-making regarding ocean acidification and its impacts. Publicly available data can be used to strengthen coastal resilience locally and regionally, reinforcing the impact of coastal management efforts. Given the diversity of scale, scope, and objectives for observing systems across the country, thoughtful and thorough data submission is critical for ocean acidification scientists to leverage the best available science to make informed decisions.

## Discussion

Determining the optimal OA observing system design is a multi-step process. The scale and scope of the observing system will depend on the intended users of the data, the local acidification processes of interest, the resource investments available to the group, and other factors. Below, we outline case studies from each regional Coastal Acidification Network that serve as examples of appropriate uses of OA observing techniques, sensor selections, and/or system designs. The case studies are not all-encompassing of potential questions to be addressed by OA observing networks, but are intended to illustrate ways that observing networks can be established effectively.

### Community sampling in Alaska

**Fig. 2:**
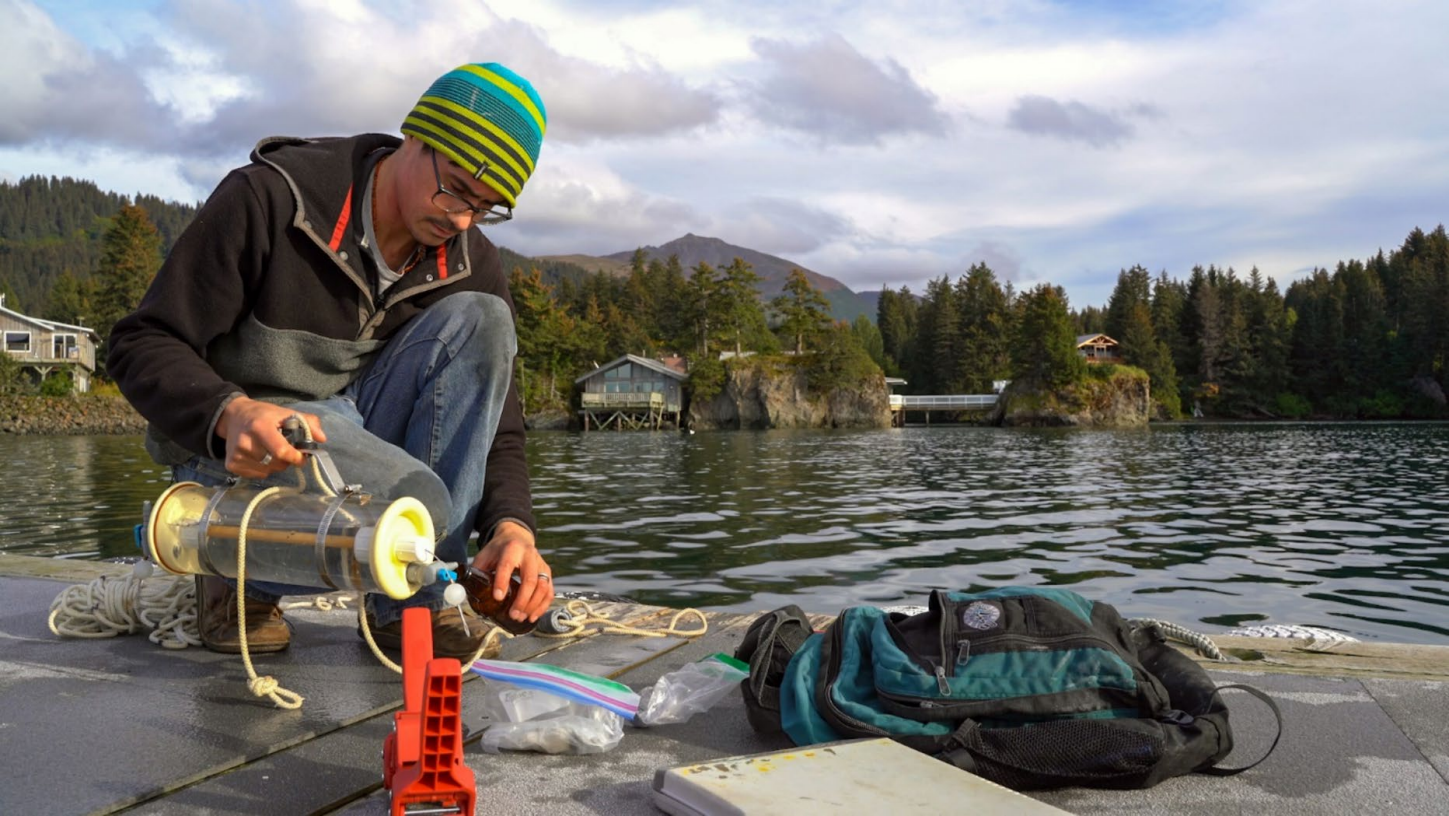
Stephen Payton, an Alaska OA Network monitoring partner at the Seldovia Village Tribe. Photo courtesy of Chugach Regional Resources Commission

In Alaska, community sampling fills critical gaps in a broader network of OA monitoring that includes moorings, underwater gliders, ship-based studies, and a state ferry. Since 2017, around 20 communities across Alaska have been involved in taking weekly water samples to develop baseline OA data. This effort is unique in the U.S. and is primarily coordinated through tribes. The water samples are used to build an understanding of current local conditions in the nearshore environment, seasonal changes, and potential natural influences. By creating a consistent time series, communities can better understand the water chemistry in areas that are important for harvesting shellfish and other species they rely on for their subsistence way of life (Fig. 2).

On a weekly basis, samplers go to their designated sampling location, usually a dock, and submerge a 5-gallon bucket into the water to collect three gallons of seawater. From the bucket, they fill a clean glass bottle with seawater, record the temperature, add the preserving agent (mercuric chloride), and cap the bottle. Water samples can be stored for weeks or months until enough bottles have been accumulated to ship to a laboratory for analysis. Three Tribal programs have taken the lead in coordinating and processing community samples in Alaska: the Alutiiq Pride Marine Institute in Seward, the Sitka Tribe of Alaska in Sitka, and the Kodiak Area Native Association in Kodiak. These entities train community samplers, ship sample collection materials, and analyze the samples using a Burke-o-Lator (Alaska Ocean Observing System, 2016), an instrument that measures temperature, salinity, pCO_2_, and TCO_2_, then uses those parameters to determine the saturation state of aragonite and pH. A consistent time series spanning multiple years is ideal for interpreting the data and understanding natural variability. Efforts are underway to analyze and interpret the data in partnership with Tribes who take the lead in reporting back to communities.

### Pacific Northwest seasonal forecasts for state, federal, and tribal resource managers

**Fig. 3:**
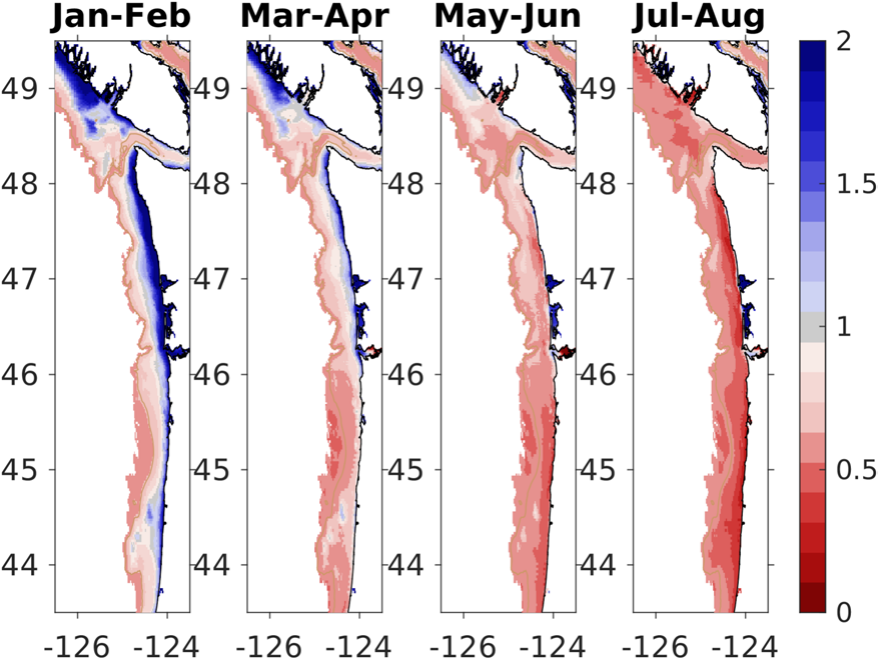
January 2025 initialized forecast from J-SCOPE provided to the Pacific Fishery Management Council. Aragonite saturation state (Ω) is expected to be more undersaturated (i.e., more corrosive, < 1) throughout the upwelling season for most of the bottom waters in the region. Surface waters are expected to be supersaturated throughout the season. See http://www.nanoos.org/products/j-scope/forecasts.php and Siedlecki et al. (2016) for more information

End user input and product goals are important to consider at the outset of establishing or utilizing a regional OA observing network. In the Pacific Northwest, the regional observing network has informed coastal resource management and tribal nation decision-making, in part through the Joint Institute for the Study of Atmosphere and Oceans Seasonal Coastal Ocean Prediction of the Ecosystem (J-SCOPE) (Northwest Association of Networked Ocean Observing Systems, n.d.; Siedlecki et al., 2016, 2023). The J-SCOPE model is a high-resolution seasonal forecast model for Washington and Oregon shelf water biogeochemistry. It was designed to integrate regional observing data relevant to end user interests in predictive OA and OA co-stressor forecasts (Fig. 3). Forecast products from J-SCOPE are key indicators for the California Current Integrated Ecosystem Assessment (CCIEA) report and fisheries habitat predictions. In each case, the end user group was included from the beginning of the product generation, with their needs identified as described in Siedlecki et al. (2023).

J-SCOPE forecast products are connected to in situ observations relied on by end users. In one example, the end user group, the Quinault Indian Nation, indicated their preference for moored assets in the benthic habitat within their fishing grounds during meetings between the J-SCOPE science team, coastal managers, and the Quinault Indian Nation. These moorings, operated by the Olympic Coast National Marine Sanctuary, the Ocean Observations Initiative (OOI), and National Oceanic and Atmospheric Administration (NOAA) and partners, observe bottom oxygen and temperature throughout most of the upwelling season and measure carbon variables through a series of regional algorithms (Alin et al., 2023). Multiple NOAA and OOI moorings are also equipped with climate-quality pCO2 at the surface and weather-quality pH sensors at depth. In 2018, after extensive observations of hypoxia and local forecasts indicating those conditions would persist through the end of the summer, the Quinault Indian Nation shortened their fishing season (Newton et al., 2022; Siedlecki et al., 2023). Because the observing system and data products were responsive to end user needs, the outputs of J-SCOPE were able to be applied in a coastal management framework.

### Policy informing biological monitoring along the California Coast

In some instances, observing system implementation is driven by policy and management recommendations. In California, many coastal ecosystems and biological processes are vulnerable to OA and hypoxia (OAH), driven by global climate change and intensified by seasonal upwelling dynamics (Breitburg et al., 2018; Chan et al., 2017; Chavez et al., 2017; Feely et al., 2008; Osborne et al., 2020). The intensification of OAH poses an increasing risk for California’s productive, biodiverse, and commercially and culturally important marine life. To address concerns, the State of California Ocean Protection Council and California Ocean Science Trust convened an expert panel to develop recommendations for feasible and impactful OAH-relevant biological observations to advance across ongoing monitoring programs (Kimball, 2021).

**Fig. 4:**
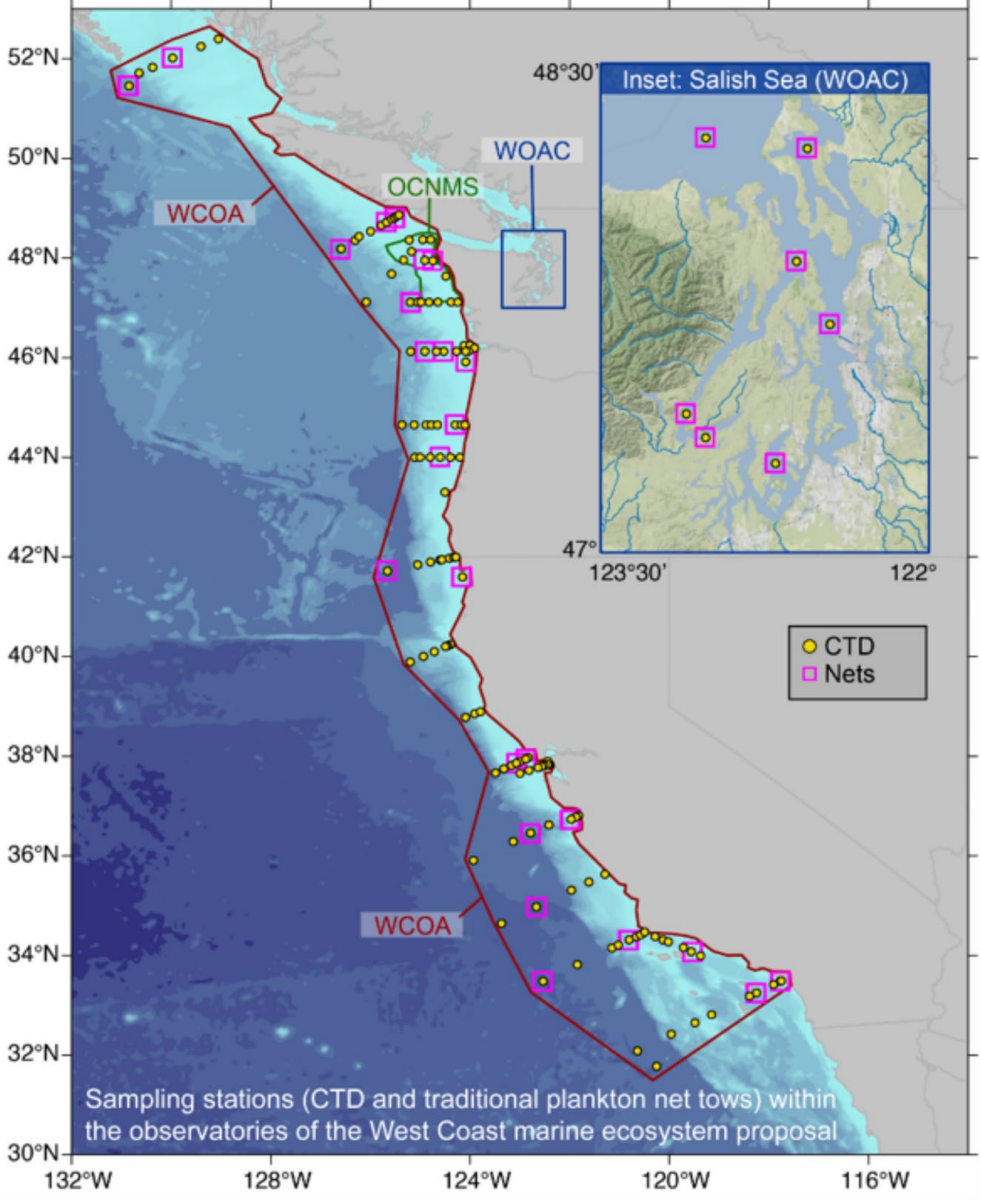
Co-located biological (nets; pink boxes) and chemical (CTD; yellow dots) sampling on the U.S. West Coast. Sampling programs shown include the West Coast Ocean Acidification cruise effort (WCOA; NOAA Pacific Marine Environmental Laboratory and NOAA Ocean Acidification Program), the Olympic Coast National Marine Sanctuary (OCNMS), and the Washington Ocean Acidification Council (WOAC). Image Credit: R. Feely, Pacific Marine Environmental Laboratory

Following global and national bodies, a key recommendation by the California Ocean Acidification and Hypoxia Science Task Force was to “better connect chemical and biological monitoring” (Kimball, 2021; Phillips et al., 2018; Weisberg et al., 2020). This lack of joint biological and chemical observations was identified by Weisberg et al. (2020) as a gap in understanding environmental impacts and attribution related to OAH. A project launched in 2021 by the State of California’s Ocean Protection Council aimed to respond to this recommendation by adding standardized biological observations onboard existing sea-going monitoring programs (e.g., California Cooperative Oceanic Fisheries Investigations, National Science Foundation Long Term Ecological Research efforts (LTER), the Southern California Coastal Water Research Program, Applied California Current Ecosystem Studies, and NOAA Ocean Acidification Program West Coast OA Cruises) to deliver priority OAH-relevant biological data (Fig. 4). Based on stakeholder needs and regional scientific expertise, they recommended that biological data streams include Dungeness crab and pteropod dissolution, harmful algal blooms (HABs), environmental DNA, zooplankton and ichthyoplankton assemblages, and krill and forage fish communities with a heavy emphasis on Dungeness crab and pteropods. These recommendations, along with suggestions about data management policies, provide a framework through which impactful OA monitoring is being pursued on the west coast.

### Tampa Bay OA monitoring and National Estuary Program partnership

Regional coastal management bodies may encounter opportunities to join forces with national-level observing networks to efficiently establish monitoring programs and ensure data usability in management problems. For example, the U.S. Geological survey (USGS) collaborated with the Environmental Protection Agency National Estuary Program (EPA-NEP), Tampa Bay Estuary Program (TBEP), University of South Florida (USF), Florida Fish and Wildlife Conservation Commission-Fish and Wildlife Research Institute (FWC-FWRI), and the University of Tampa (UT) to initiate the first OA monitoring program in Tampa Bay (Rosenau et al., 2021). The goals were to (1) quantify short-term (daily to monthly) and long-term (seasonal to interannual) changes in carbonate chemistry and ecosystem exposure to low pH conditions; (2) identify physical, biological, and chemical drivers of coastal acidification; and (3) examine the potential for seagrass to buffer acidification in Tampa Bay as a benefit of long-term seagrass restoration efforts.

**Fig. 5:**
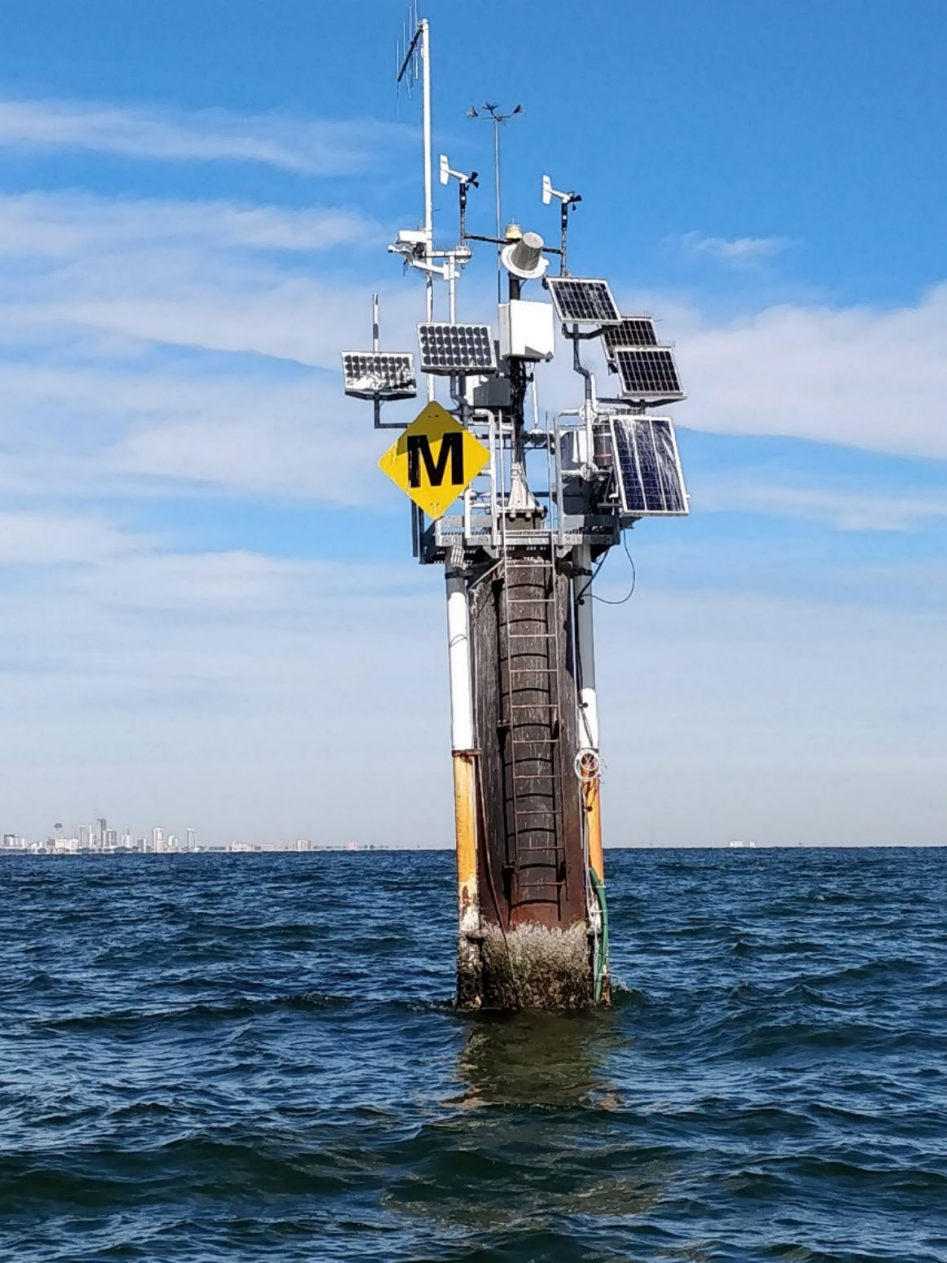
Middle Tampa Bay LOBO OCS location, showing the variety of in situ sensor packages found at each station

As part of this effort, autonomous sensor packages were deployed to measure hourly changes in carbonate system parameters in Tampa Bay and the near-coastal Gulf of America environment. Results from a discrete sampling pilot study (Yates et al., 2016) informed the placement of sensor monitoring systems. In situ sensor packages (Ocean Carbon Systems (OCS) integrated Seabird SeapHOx instruments, Pro-Oceanus CO2-Pros, and EcoPar sensors) were deployed to measure seawater pH_T_, CO_2_, dissolved oxygen, light climate (photosynthetically active radiation, or PAR), and physical water conditions (conductivity, temperature, and depth) (Fig. 5). A key limitation for deploying sensors in estuarine environments is the need for frequent field expeditions to clean biofouling and debris from sensor systems. Data gaps occur when sensors must be removed for intensive cleaning or repair, and using duplicate sensors to swap out instruments is costly.

The near real-time data from these sensors allow researchers to identify estuarine influences on acidification conditions (Yates et al., 2023). The data are publicly available at http://tampabay.loboviz.com/. Monitoring results are used to evaluate the role of seagrass in buffering impacts from coastal acidification, freshwater discharge events related to storms and local mining water release events, and changes in the carbonate system associated with harmful algal blooms. This information feeds into the broader NEP network to inform coastal management decisions at a national level.

### Subsurface automated samplers in the NOAA National Coral Reef Monitoring Program in Puerto Rico and US Virgin Islands

**Fig. 6:**
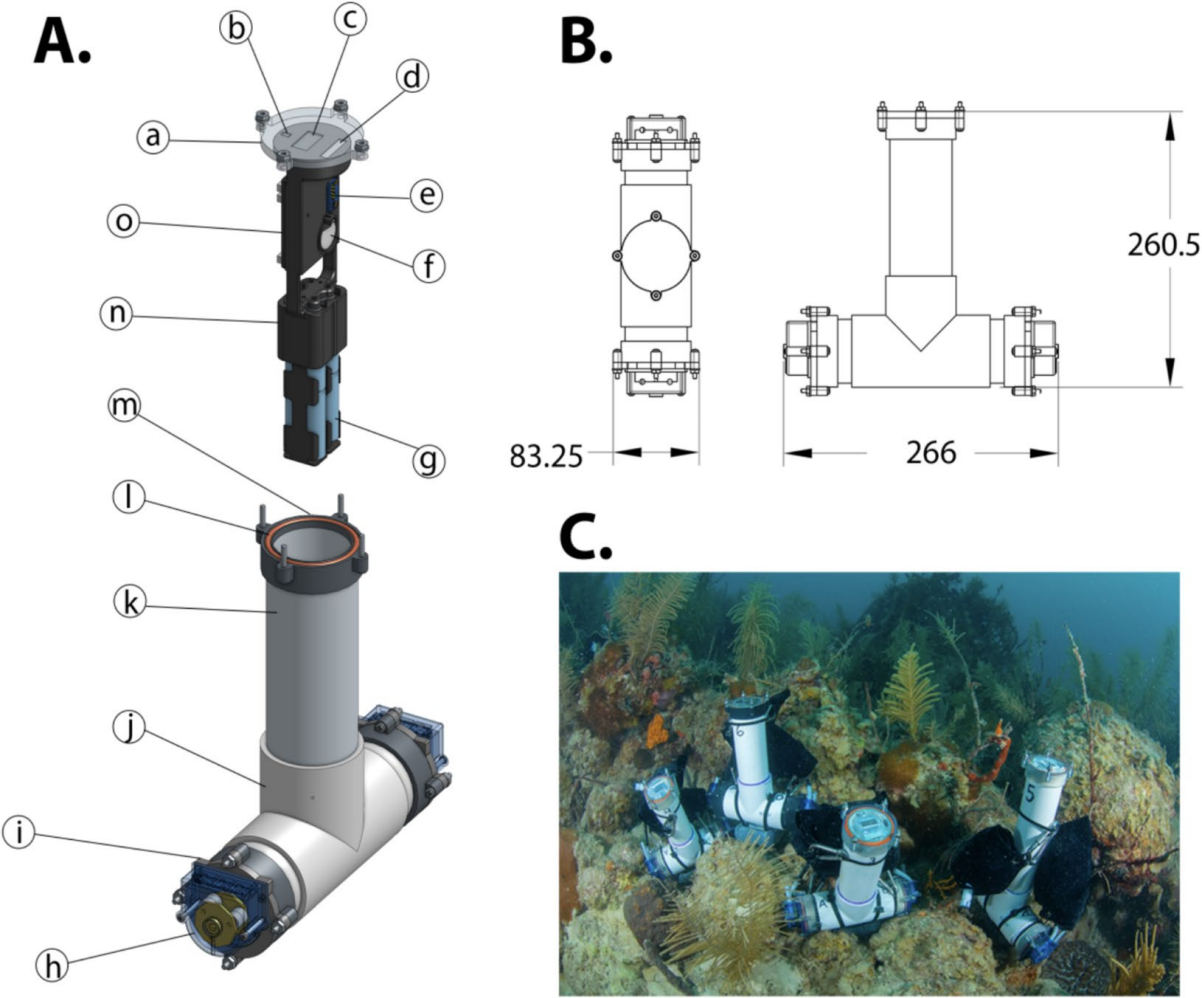
**A** Three-dimensional rendering of the Subsurface Automated Sampler highlighting individual parts available for 3D printing and/or purchase: (a) acrylic faceplate, (b) infrared detector, (c) OLED display, (d) magnetic switch), (e) temperature sensor, (f) coin cell battery for onboard clock, (g) AA battery packs, (h) peristaltic pump head, (i) waterproof motor faceplate, (j) PVC tee., (k) PVC pipe, (l) waterproof end cap flange, (m) O-ring, (n) internal armature, and (o) microcontroller and circuit board. **B** Dimensional (mm) drawing of external SAS housing. **C** Four SAS deployed as part of NOAA’s National Coral Reef Monitoring Program (photo credit K. Davidson and ANGARI Foundation). This figure was originally created for and published in Enochs et al., 2020, and is recreated here with permission from the copyright holder

Carbonate chemistry parameters can be challenging to collect due to instrumentation and field time costs, variable ocean conditions for sampling, and difficult field deployments (Enochs et al., 2020). To alleviate some of these barriers, researchers at the NOAA Atlantic Oceanographic and Meteorological Laboratory (AOML) developed a subsurface automated sampler (SAS) to collect discrete water samples at coral reef sites (Fig. 6). Coral reefs are important ecosystems for the Caribbean region that provide habitat, food security, and coastal protection. The National Coral Reef Monitoring Program (NCRMP), funded by the NOAA Coral Reef Conservation Program, is a long-term monitoring effort conducted every two to three years to collect biological, climate, and socioeconomic data across reef sites in the United States to track changing conditions and overall ecosystem health. The NCRMP partners with local state governments, academic institutions, non-governmental organizations, and NOAA laboratories to conduct the multidisciplinary research.

Three NCRMP Caribbean data collection sites operate AOML’s SAS water samplers. The samplers are open source, with publicly available build instructions, sampling components, and software. The SAS are low-cost discrete water samplers that take samples every three hours at a reef site over the course of seven days. With the frequency of sampling, SAS can be used to determine the diurnal fluctuations of a coral reef’s carbonate chemistry parameters for a total of three days. Data from the carbonate chemistry variability of coral reef systems can be used for modeling predictions of future ecosystem responses to acidified ocean conditions. Some limitations for SAS include long-term field deployment challenges with erosion of equipment. These systems are best for short-term deployments (Enochs et al., 2020). Overall, accurate characterization of water chemistry at coral reef sites is a challenge for the research community. The SAS provides a monitoring tool that allows researchers to analyze coral reef carbonate chemistry over time and space as a low-cost field instrument.

### OA observing and coastal management in the Northeast

The Northeast region has strong seasonal variability, freshwater influences and high productivity, particularly near the coast (Gledhill et al., 2015; Siedlecki et al., 2021). States in the Northeast region have supported OA commissions or task forces since Maine’s initial OA commission in 2014. All of them recommend enhanced OA monitoring, especially in the context of other marine stresses such as eutrophication, hypoxia and HABs. Commission reports stress uncertainty in OA impacts, which can be mitigated by observing systems (Maine State Legislature, 2015; Massachusetts OA Commission, 2021).

#### Monitoring OA in long island sound

The Long Island Sound Water Quality Monitoring Program is an EPA-sponsored National Estuary Program (NEP). This monitoring program was initiated in 1985 to gather data for hypoxia and helped to set a total maximum daily load for nitrogen (Fitzpatrick et al., 2003). In 2011, the Connecticut Department of Energy and Environmental Protection began collecting pH data in Long Island Sound in addition to the standard NEP monitoring. In 2014, the Interstate Environmental Commission additionally began collecting pH data in the summer, which expanded to year-round pH sampling in 2019. In 2018, two buoys were established to provide pCO2 and pH data in the western and central Long Island Sound. Further budget requests in 2022 included OA monitoring and a new site at Oyster Bay to collect DIC, TA and pH, and a Quality Assurance Plan for the region is being finalized. Rather than OA being the primary monitoring objective at the outset of the sampling program, these OA monitoring systems were added to ongoing water quality monitoring programs. If the OA monitoring network were developed from scratch, these may not have been the ideal observing locations. However, it was a priority to co-locate OA measurements with the longer-term water quality measurements that provide oceanographic context. These measurements allow scientists to better understand coastal acidification and its controls; drivers; interactions with other stressors such as warming, hypoxia, eutrophication, and HABs; and its impacts on important species and habitats. Eventually, the program would like to establish a Long Island Sound specific value for aragonite saturation state to better understand impacts on shellfish species, and to integrate coastal acidification monitoring data into existing modeling efforts for long-term climate change projections. The monitoring network as it has been established helps to target those goals.

#### OA data informing state of the ecosystem reports

**Fig. 7:**
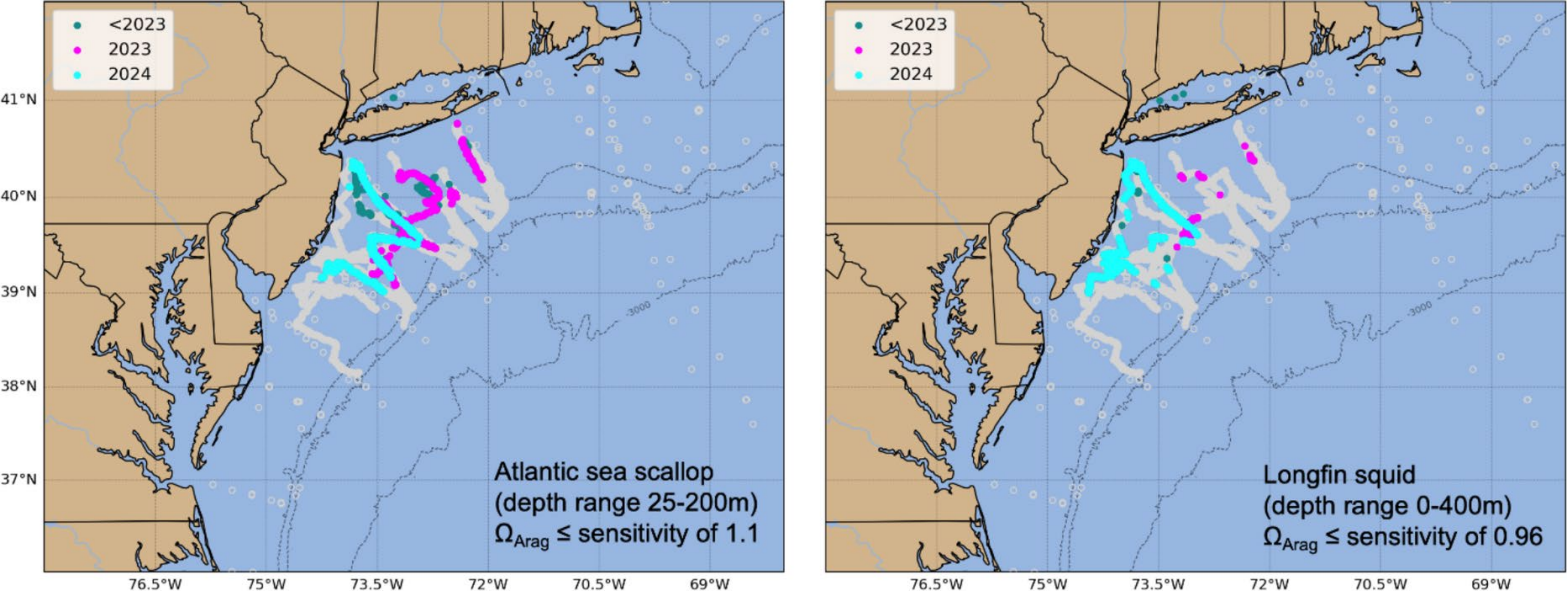
Locations where bottom aragonite saturation state in summer (June–August) was at or below laboratory-derived sensitivity levels for Atlantic Sea scallop and Longfin squid from 2007 to 2022 (dark cyan), in 2023 (magenta), and in 2024 (cyan). Gray circles indicate locations where bottom aragonite saturation state values were above the species sensitivity value. This figure was created by Grace Saba and Lori Garzio of Rutgers, the State University of New Jersey, using OA observing systems in the Mid-Atlantic. It appears in the 2025 State of the Ecosystem reports for the Mid-Atlantic States (National Marine Fisheries Service, 2025)

The National Marine Fisheries Northeast Fisheries Science Center conducts a large-scale annual assessment for the entire east coast for their State of the Ecosystem reports to the New England Fisheries Management Council and the Mid-Atlantic Fisheries Management Council. The ecosystem reports are part of a larger Integrated Ecosystem Assessment process, which integrates physical, biological, economic, and social components and allows managers to balance trade-offs and determine what is more likely to achieve their desired goals. New England and the Mid-Atlantic have slightly different approaches to IEAs, but each uses a structured decision-making process that starts with prioritizing interactions and developing questions, developing a conceptual model, and modeling in a Management Strategy Evaluation process to determine how to deal with multiple species management. In 2023, NOAA’s State of the Ecosystem Report included OA conditions for the first time (Fig. 7). OA observatories in the fishery regions, including underwater gliders equipped with pH sensors and NOAA-sponsored East Coast Ocean Acidification and Ecological Monitoring cruises, are used to highlight areas where scallops may be exposed to harmful OA conditions each year. The inclusion of synthesized data products increases awareness of OA in the region.

### Integrated observing systems in the southeast

Across broad regions that consist of varied ecosystems and OA drivers, there may not be a one-size-fits-all solution to OA observing. The Georgia coast is a prime example, with shelf waters of the South Atlantic Bight (SAB) and coastal marshes in close proximity. The SAB experiences a confluence of coastal processes (Xue et al., 2017), shelf dynamics (Signorini & McClain, 2007), and Gulf Stream influences (Castelao, 2014). Dynamic observing efforts are needed to understand the physical processes that contribute to SAB acidification and the biological impacts across the region.

**Fig. 8:**
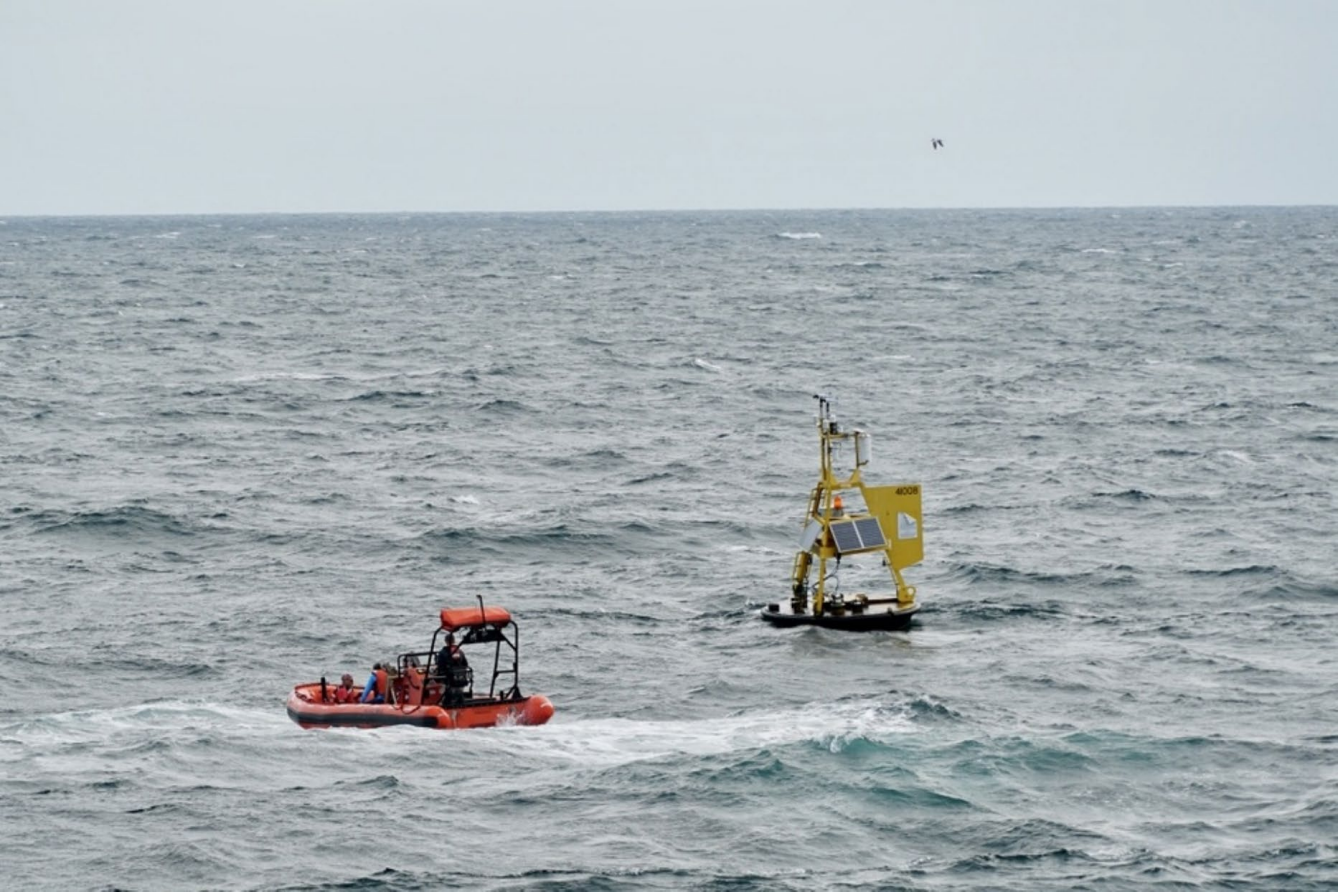
The Gray’s Reef MAPCO2 buoy (right), located in Gray’s Reef National Marine Sanctuary, is visited via small boat operations (left) from the East Coast Ocean Acidification cruise (ECOA-3) in 2022. Scientists aboard the orange vessel took water samples close to the buoy, allowing for cross-calibration of OA data. Photo credit: NOAA Ocean Acidification Program

The Gray’s Reef mooring, located in the coastal SAB approximately 23 nautical miles off the coast of Savannah, Georgia, has measured climate-quality surface *p*CO_2_ since summer of 2006. In the same region in 2014, six cruises conducted by the Georgia Coastal Ecosystems LTER (GCE) collected discrete carbonate chemistry measurements and underway cruise *p*CO_2_ within the marshes and out onto the SAB shelf. These and other efforts, including a 2016 marsh to shelf cruise and quarterly sampling within the GCE domain (https://gce-lter.marsci.uga.edu/) for DIC and TA, have shown that the SAB shelf is well buffered due to the high TA and salinity of the sub-tropical waters (Egleston et al., 2010), yet incremental ocean acidification has still been observed there over the past two decades (Reimer et al., 2017a, 2017b). On the other hand, inter-coastal and marsh waters along the Georgia coast could be hotspots for acidification due to freshwater runoff and high productivity and subsequent respiration (Letourneau & Medeiros, 2019; Reimer et al., 2024). The combined discrete, underway, and moored autonomous sampling for carbonate chemistry parameters in the SAB exemplify how multiple monitoring methods can be combined to depict the state of acidification in a dynamic environment and across the terrestrial-coastal-ocean continuum (Fig. 8).

### CSI:Oyster—a novel Mid-Atlantic community science program

In Mid-Atlantic estuaries, coastal acidification is influenced by local drivers (e.g., freshwater input and eutrophication), creating highly variable carbonate chemistry conditions across spatial and temporal scales. The Chesapeake Bay is prone to episodic acidification events (with pH as low as 7.3) which can adversely impact Eastern oysters, *Crassostrea virginica*, an economically and ecologically important species in the region (Cai et al., 2021). In particular, larval stages of *C. virginica* are vulnerable to acidification. Coupling biological and chemical monitoring to determine how site-specific coastal acidification conditions affect oyster growth and development is key to supporting the rapidly expanding aquaculture industry and the ongoing living shoreline and reef restoration efforts in the Chesapeake Bay.

CSI:Oyster is a high school and community college science program that engages students in field data collection. Led by Virginia Institute of Marine Science researcher Emily Rivest with funding from Dominion Foundation Environmental Education and Stewardship Grant, NOAA OAP, and the National Science Foundation, this community program offers an opportunity for the students to learn while simultaneously gathering information to improve understanding of local trends in acidification, duration of oyster exposure to low pH conditions, and subsequent impacts on oyster ecology. Students deploy juvenile *Crassostrea virginica* oysters in mesh bags at 13 sites along tidal tributaries in the upper and lower Chesapeake Bay. Growth, survival, and water quality are monitored every two to four weeks throughout the summer.

**Fig. 9:**
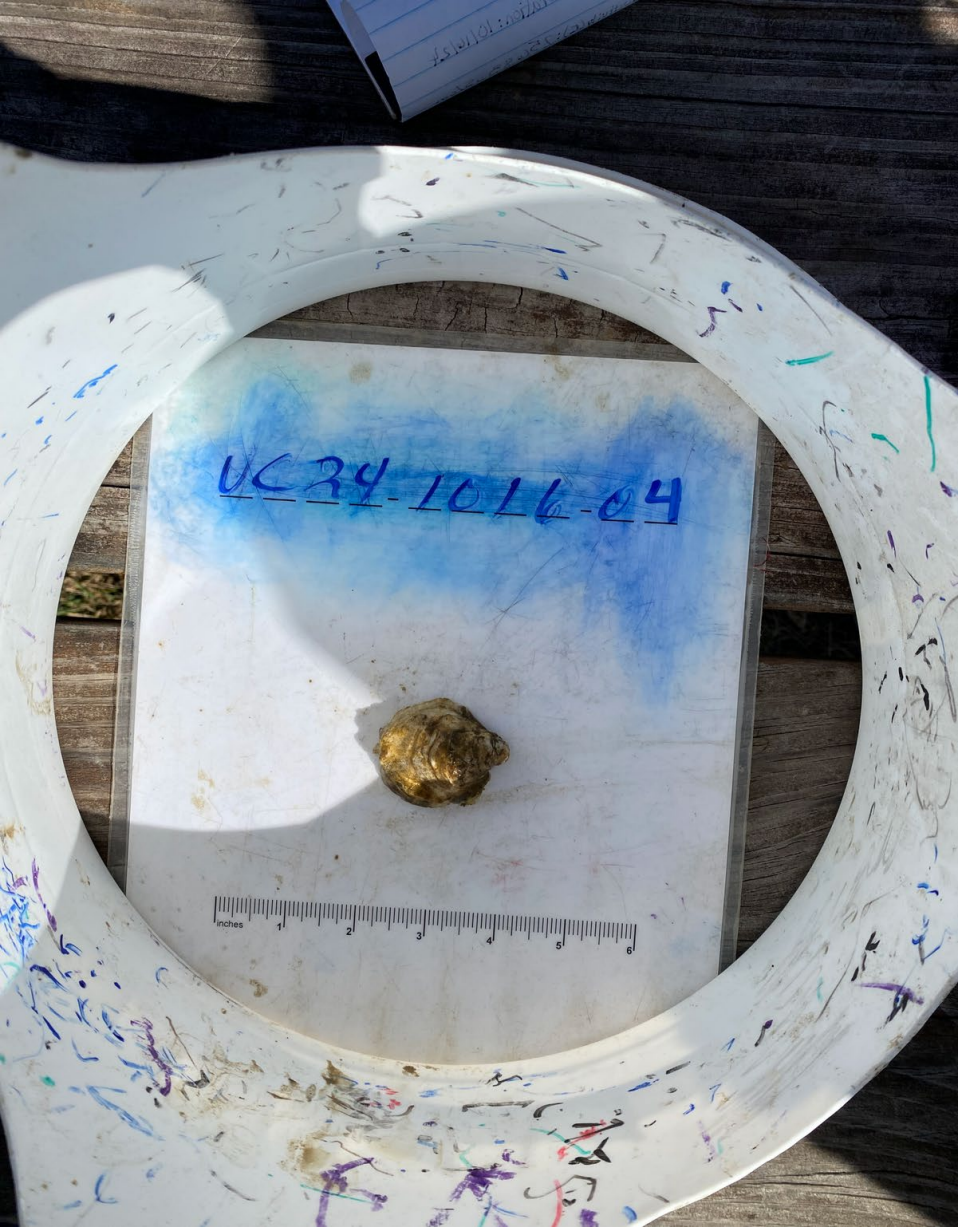
After oyster shells have been measured with calipers to determine length and width, students capture photos of each oyster using their phone and the photo bucket shown above. The images are analyzed with ImageJ software in the laboratory. Caliper measurements are compared to the ImageJ analysis for accuracy and data quality control. Photo Credit: Sara Chaves Beam, Instructor, Marine and Environmental Sciences, Chesapeake Bay Governor’s School for Marine and Environmental Science- Glenns Campus

**Fig. 10:**
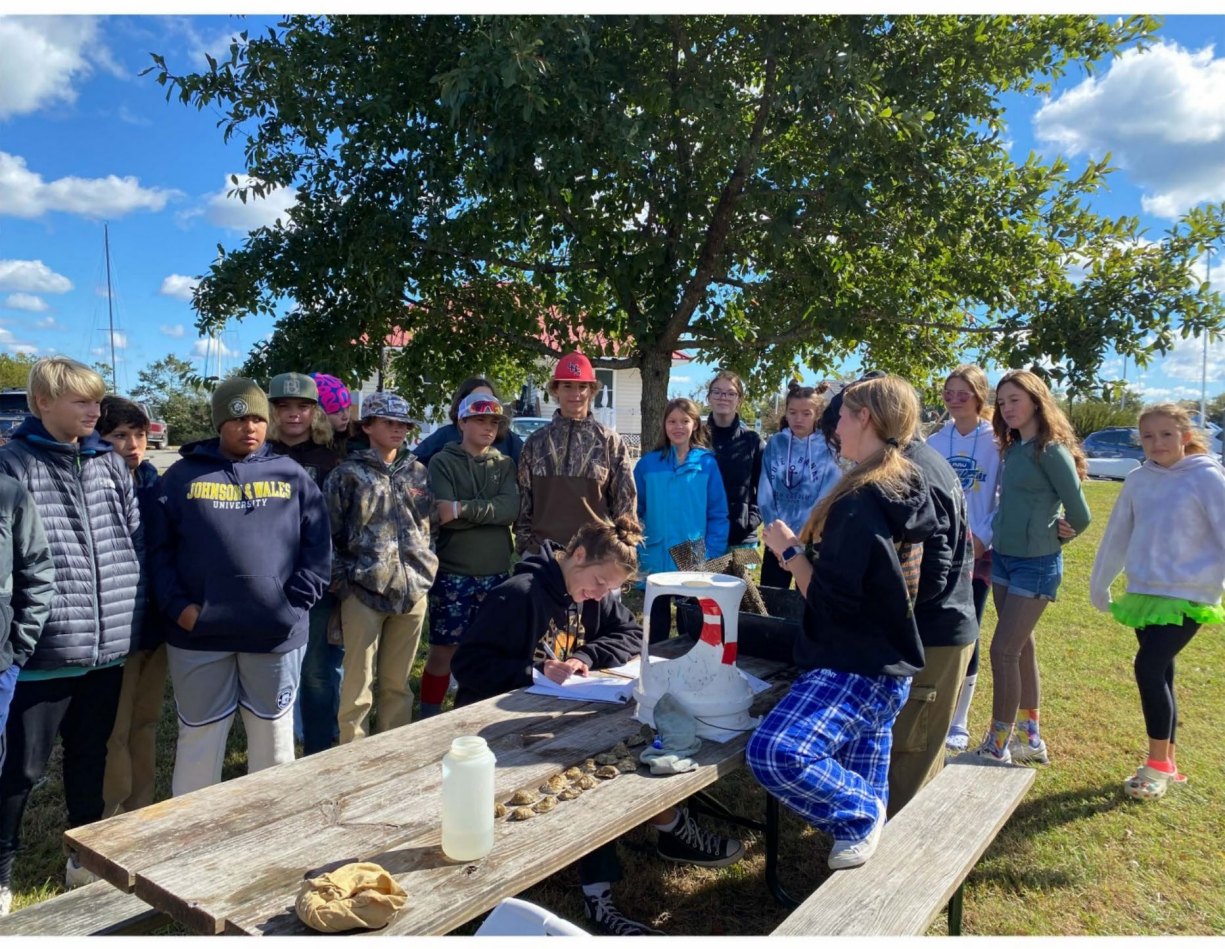
At a field sampling site in Virginia, a student demonstrates how calipers are used to measure oyster shell length from hinge to bill. After recording the measurements on a data sheet, students use a photo bucket to take images of each oyster. Photo Credit: Sara Beam, Sara Chaves Beam, Instructor, Marine and Environmental Sciences, Chesapeake Bay Governor’s School for Marine and Environmental Science- Glenns Campus

Oyster growth is measured with calipers and documented with photographs; a growth time series is developed with ImageJ photo analysis software (Fig. 9). Water temperature, salinity, dissolved oxygen, and pH data are collected with a multiparameter handheld meter. Bottle samples are preserved for laboratory analysis of pH and TA using standard best practices. Calcite saturation state (Ωca) is calculated for each site. With over five years of data collected, students are building a baseline of local acidification conditions and documenting the impacts of acidification on oyster shell growth (Fig. 10). CSI:Oyster data has been integral for calculating alkalinity-salinity relationships, particularly for regions with limited alkalinity data like the tidal tributaries in the lower Chesapeake Bay, and for validating models of when and where OA thresholds are likely to occur (Czajka, 2024; Czajka et al., in prep; Da, 2023; Da et al., 2024).

### Conclusions

Design and implementation of an OA observing network is a multi-step process that differs between regions, program capacities, needs of interested parties, and funding availability. OA observing networks can maximize project benefits by utilizing end-to-end design processes to ensure the network is achievable, sustainable, and will provide valuable technical assistance for those most dependent on or interested in the ecosystem. Utilizing the existing CANs, local organizations, community members, and other regional interest groups can be brought into the design process for new monitoring programs. Collaboration with local experts to identify major scientific interests related to water quality and climate change impacts can help ensure the impact of the monitoring network. Co-production of knowledge and information products is critical for an end-to-end, iterative design process. As carbonate system parameters and sensors are chosen, it is important to keep the end goals of the program in mind, consider which data will be most useful to the end user, and have plans in place to ensure accessibility of data and data products. As demonstrated by the regional case studies provided here, establishment of an OA monitoring network utilizing CAN expertise is achievable across broad scales of ecosystem dynamics, organizational capacity, funding availability, and local interests.

No new or original data was produced as a result of this work. This work was not produced under any specific funding award, but was prepared by representatives of Regional U.S. Coastal Acidification Networks, which are grassroots organizations sponsored in part by the National Oceanic and Atmospheric Administration’s Ocean Acidification Program (ROR 02bfn4816).

## Supplementary Information

Below is the link to the electronic supplementary material.

Supplementary file1 (PDF 230 kb)

## Data Availability

No datasets were generated or analysed during the current study.
