# Supplementary material for: Management considerations for establishing a coastal acidification monitoring system from U.S. Coastal Acidification Networks
Source: Environ Monit Assess. 2025 Aug 7;197(9):990. doi: 10.1007/s10661-025-14434-3 (PMC12331854; doi:10.1007/s10661-025-14434-3)
Supplement: Supplementary file 1 — Supplementary file1 (PDF 230 kb) [file 10661_2025_14434_MOESM1_ESM.pdf]

Article Title: Management considerations for establishing a coastal acidification monitoring system from U.S. Coastal Acidification Networks

Author Names: Elizabeth K. Wright-Fairbanks[1,2], Natalie Lord[3], Darcy Dugan[4,5], Kaitlin Goldsmith[1,2], Emily R. Hall[6,7], Alex Harper[8,9,10], Janet J Reimer[7,11,12] , Samantha Siedlecki[13,14], Elizabeth J. Turner[14], Jennifer Vreeland-Dawson[2,15], Kirstin Wakefield[12,16], Kimberly K. Yates[15,17]

Affiliations:
[1]University Corporation for Atmospheric Research, Boulder, CO, United States
[2]National Oceanic and Atmospheric Administration, Silver Spring, MD, United States
[3]University of New Hampshire, Durham, NH, United States
[4]Alaska Ocean Observing System, Anchorage, AK, United States
[5]Alaska Ocean Acidification Network, Anchorage, AK, United States
[6]Mote Marine Laboratory, Sarasota, FL, United States
[7]Southeast Ocean and Coastal Acidification Network, Sarasota, FL, United States
[8]California State Polytechnic University, Humboldt, CA, United States
[9]California Current Acidification Network, San Diego, CA, United States
[10]California Sea Grant, San Diego, CA, United States
[11]Mid-Atlantic Regional Council on the Ocean, Washington, DC, United States
[12]Mid-Atlantic Coastal Acidification Network, Washington, DC, United States
[13]University of Connecticut, Groton, CT, United States
[14]Northeast Coastal Acidification Network, Portland, ME, United States
[15]Gulf of America Coastal Acidification Network, College Station, TX, United States
[16]Mid-Atlantic Regional Coastal Ocean Observing System, Washington, DC, United States
[17]Gulf of America Coastal Ocean Observing System, College Station, TX, United States

Corresponding Author: Elizabeth K. Wright-Fairbanks; lwfairbanks@ucar.edu; 1315 East-West Highway, Silver Spring, Maryland 20910; 978-604-2875; ORCiD: 0000-0003-1265-6616

Table S-1. References describing methods and standard operating procedures (SOPs) for observation networks, OA research practices, and measurement of carbonate system parameters.

**Method or SOP**	**Reference**
Global ocean observing network development and requirements	Newton et al., 2015
Best practices for OA research and data reporting	Riebesell et al., 2011
Assessing trends in ocean acidification time series	Sutton et al., 2022
Water sampling procedures for carbonate system, including:<br>• Determination of DIC in seawater<br>• Determination of TA in seawater<br>• Determination of *p*CO_2 in air<br>• Determination of pH in seawater<br>• Determination of DOC and total dissolved nitrogen<br>• Gravimetric calibrations: volume of gas loop, or other volumetric containers<br>• Preparation of sodium carbonate solutions for calibration of coulometric DIC measurements<br>• Statistical techniques, calculations, and corrections	Dickson et al., 2007
Determination of carbonate ion concentrations in seawater	Sharp and Byrne, 2019.
Calibration of glass pH electrodes	Martell-Bonet and Byrne, 2020
Data Quality Control methods and best practices	Integrated Ocean Observing System, 2019; Environmental Protection Agency, 2021; Jiang et al., 2022.

Table S-2. Sensor examples and specifications. Modified and updated from Rosenau et al. 2021. Readers should refer to instrument manufacturer guidelines for the most up-to-date information.

**Instrument**	**Parameter**	**Accuracy**	**Precision**	**Equilibration Time**	**Range**	**Calibration Frequency**
SunBurst SAMI-CO_2	pCO_2	± 3 matm	<1 matm	5 min.	150–700 matm	Annual (Sunburst Sensors *a*)
SunBurst SAMI-pH	pH_T	0.003	<0.001	3 min.	7-9	Annual (Sunburst Sensors *b*)
Pro-Oceanus CO_2-Pro CV	pCO_2	0.5% of value	0.01 ppm	< 1min.	0–10,000	Annual (Pro-Oceanus)
MAPCO_2	pCO_2	2 mmol mol^{-1}	<0.6 mmol mol^{-1}	10 min.	0–800 mmol mol^{-1}	12-18 months (Sutton et al., 2014; Sensor and System Solutions)
Sea-Bird SeapHOx	pH_T	0.05	0.004	< 1min.	6.5–9	Annual (Sea-Bird Scientific, 2024)
	DO	3 mmol kg^{-1}	1 mmol kg^{-1}	< 1min.	120% of saturation	
	Temp	0.002 °C	0.002 °C	< 1min.	-5 to 45 °C	
Seabird SeaFET v2	pH	0.05	0.004	< 1min.	6.5–9	
Aanderaa Oxygen Optode	DO	< 8 mM	<0.5% yr^{-1}	< 1min.	0–1,000 mM	Annual (Aanderaa, 2017)

Table S-3. Examples of programs and project applications of sensor and discrete monitoring approaches.

**Program/Project**	**Data Type**	**Region**	**Data Access Platform or Informational Link**
GOMECC (2007, 2012, 2017, 2021)	Discrete/cruise: water column DIC, TA, pH, pCO_2, CO_3^{2-}, DO, nutrients, chl and surface pCO_2,	Gulf of America	National Centers for Environmental Information Ocean Carbon and Acidification Data System: https://www.ncei.noaa.gov/access/ocean-carbon-data-system/
nGOMx Acidification (2017, 2018, 2019)	Discrete/cruise: water column DIC, TA, pH, DO and	Gulf of America Shelf	Biological and Chemical Oceanography Data Management Office: https://doi.org/10.26008/1912/bco-dmo.831523.1
nGOMx Bottom Water (2006-2017)	Discrete/cruise: bottom water DIC and TA	Gulf of America Shelf	Biological and Chemical Oceanography Data Management Office: https://doi.org/10.26008/1912/bco-dmo.818773.1
nGOMx Shelfwide Hypoxia Cruise (2010, 2011, 2012, 2013, 2014, 2015, 2016)	Discrete/cruise: water column DIC, TA, pH	Gulf of America Shelf	Gulf Science Data Repository: https://doi.org/10.7266/N7Z899TR https://doi.org/10.7266/N7513WM8 https://doi.org/10.7266/N78913VT https://doi.org/10.7266/N7000046 https://doi.org/10.7266/N73R0QXQ https://doi.org/10.7266/N77H1GM0 https://doi.org/10.7266/N7GF0S2N
Coastal Louisiana OA Mooring (2017-present)	Sensor: surface and atmospheric CO_2 and surface water pH	Gulf of America Shelf	https://www.pmel.noaa.gov/co2/story/Coastal+LA
Cheeca Rocks Coral Reef OA Mooring (2011-present)	Sensor: surface and atmospheric CO_2 and surface water pH	Southeast Region Florida Shelf	https://www.pmel.noaa.gov/co2/story/Cheeca+Rocks

Tampa Bay Estuary and Eastern Gulf of America Mooring (2018-present)	Sensor/discrete: surface pH, pCO_2, DO; discrete mid-water column TA, DIC, pH	Gulf of America Florida Estuaries	http://tampabay.loboviz.com/ https://coastal.er.usgs.gov/data-release/doi-P91T185R/ https://doi.org/10.5066/P9HS7ZV0 https://coastal.er.usgs.gov/data-release/doi-P9BAFC7L/
Texas Estuarine Acidification Sites Mooring (2016-2017)	Sensor: surface pH and pCO_2	Gulf of America	National Centers for Environmental Information: https://doi.org/10.25921/dkg3-1989
Estuarine Acidification Sites (2014-2017 & 2018-2020)	Discrete: water column TA, DIC, pH	Gulf of America Texas Estuaries	Biological and Chemical Oceanography Data Management Office: http://doi.org/10.26008/1912/bco-dmo.835227.1 http://doi.org/10.1575/1912/bco-dmo.784673.1
Flower Garden Banks Seasonal Sampling (2013-2016)	Discrete: water column TA, DIC, pH	Gulf of America Shelf	Gulf Science Data Repository: http://doi.org/10.7266/N7G15Z9M
TAMU OA Cruises (2015-2019)	Discrete: water column TA, DIC, dissolved inorganic nutrients, DO	Gulf of America Shelf	Biological and Chemical Oceanography Data Management Office:https://www.bco-dmo.org/dataset/787575
NOAA East Coast Ocean Acidification Cruise	Discrete/cruise: multiple variables	Northeast: East Coast	National Centers for Environmental Information Ocean Carbon and Acidification Data System: https://www.ncei.noaa.gov/access/ocean-carbon-data-system/
EPA National Estuary Program Ocean Acidification Monitoring	Sensors: pH, pCO_2, DO	All U.S. regions	Rosenau et al., 2021
NOAA's National Ocean Acidification Monitoring Network (NOA-ON) Buoys	$MAPCO_2$	All U.S. regions	NOAA Ocean Acidificaiton Program: https://oceanacidification.noaa.gov/ocean-acidification-research/ocean-acidification-monitoring/#buoys-moorings NOAA PMEL:

			https://www.pmel.noaa.gov/co2/story/Coastal+Moorings
Friends of Casco Bay	Sensors: water temperature, salinity, DO, chl, turbidity and pH. Discrete: pH and TA	3 mooring stations	https://www.cascobay.org/our-work/science/continuous-monitoring-stations/
Gray's Reef National Marine Sanctuary Mooring	Sensor: pH, pCO_2	Southeast Region	https://www.pmel.noaa.gov/co2/story/Grays+Reef
Alaska Ocean Acidification Observing System	Moored sensors, ship-based sampling, community sampling	Alaska	https://aoan.aoos.org/alaska-oa-data-list-and-links/
Alaska Marine Highway Ferry	Ship-based monitoring	Between Bellingham, WA, and Skagway, AK, along the Seward and Kodiak Lines in the Gulf of Alaska, and within Cook Inlet	https://aoan.aoos.org/alaska-oa-data-list-and-links/
WRI-Mote Cooperative Red Tide Monitoring and Research Program	Discrete cruise: TA, DIC, pHT, DOC, nutrients	Gulf of America, west Florida coasts and estuaries	https://myfwc.com/research/redtide/monitoring/database/
